# Understanding the genetic basis of heat stress tolerance in wheat (*Triticum aestivum* L.) through genome‐wide association studies

**DOI:** 10.1002/tpg2.70071

**Published:** 2025-07-09

**Authors:** Santosh Gudi, Jatinder Singh, Harsimardeep Gill, Sunish Sehgal, Justin D. Faris, Upinder Gill, Rajeev Gupta

**Affiliations:** ^1^ Department of Plant Pathology North Dakota State University Fargo North Dakota USA; ^2^ Department of Agronomy, Horticulture, and Plant Science South Dakota State University Brookings South Dakota USA; ^3^ Edward T. Schafer Agricultural Research Center USDA‐ARS Fargo North Dakota USA

## Abstract

Heat stress can reduce the production potential of wheat (*Triticum aestivum* L.) by affecting the various developmental stages of wheat including the seedling stage. Understanding the genetic basis of heat stress tolerance can help in breeding resilient wheat cultivars with improved productivity. Here, evaluation of a diverse panel of spring wheat landraces and cultivars under non‐heat stress (23°C) and heat stress (36°C) treatments in a controlled environment revealed large phenotypic and genetic variations. Heat stress negatively affected all seedling traits with the maximum reduction in root length (85.6%) and the least reduction in coleoptile length (15.44%). Moreover, based on seedling performance, we identified six highly heat tolerant (PI 366905, Kzyl Sark, Rang, Perico S, Bohr Gamh, and PI 620689) and six highly heat susceptible (CItr 17470, CItr 13270, Coeruleum, Shashi, Hallany, and Currawa) genotypes. Genome‐wide association analysis using 302,524 single nucleotide polymorphisms identified 23 marker‐trait associations (MTAs), of which 16 were associated with various seedling traits under heat stress. Gene annotation and expression analysis indicated 35 differentially expressed genes, of which 13 were considered as high‐confidence genes with functional relevance to heat stress including protein kinase, basic‐leucine zipper, UDP‐glucosyltransferase, pyrophosphate‐energized proton pump, fatty acid hydroxylase, and other classes of proteins. The MTAs and candidate genes identified in this study hold promise for developing heat‐resilient wheat cultivars through the selection of favorable alleles with gene‐specific molecular markers.

## Core Ideas

- Evaluating global spring wheat diversity panel identified highly heat‐tolerant lines.
- A genome‐wide association studies (GWAS) identified 23 marker‐trait associations (MTAs) associated with different seedling traits in wheat.
- Expression analysis pinpointed candidate genes (CGs) putatively involved in heat stress tolerance.

**Abbreviations**
BLUE: best linear unbiased estimates
CGs: candidate genes
GWAS: genome‐wide association studies
MLM: mixed linear model
MTAs: marker‐trait associations
QTLs: quantitative trait loci

## 1 INTRODUCTION

Wheat (*Triticum aestivum* L.) is the third most grown food crop of the United States following corn and soybean with an annual production of 1.65 billion bushels from 47.2 million acres (https://www.nass.usda.gov/). North Dakota is the second largest producer of wheat in the United States with production totaling 307 million bushels during 2023–2024 (https://www.ndwheat.com). However, global wheat production is constrained by several biotic (such as rust, Fusarium head blight, powdery mildew, tan spot, etc.) and abiotic stresses (such as heat, drought, salinity, ion toxicity, etc.) (Rajamanickam et al., 2024; Gudi et al., 2025). Heat stress is the major abiotic stress that negatively impacts wheat growth at various developmental stages. For instance, heat stress at the early developmental stages affects seed germination and seedling establishment (Farhad et al., 2023; Gudi, Halladakeri, et al., 2024). Heat stress reduces the photosynthetic efficiency, respiration, stomatal conductance, net assimilation rate, and water use efficiency (Coast et al., 2024; Gudi et al., 2023). Additionally, heat stress ruptures cell membranes and inactivates key enzymes involved in growth and development, thereby reducing photo‐assimilation, shoot and root biomass production, and tiller number per unit area (Djanaguiraman et al., 2020; Dwivedi et al., 2024; Gudi, Jain, et al., 2024). Heat stress also results in the increased production of reactive oxygen species, which are detrimental to cellular metabolisms, such as damage to nucleic acids and membrane peroxidation (Abasi et al., 2024; Bita & Gerats, 2013).

Additionally, in the later developmental stages, heat stress leads to pollen sterility and subsequently decreases grain number per spike, grain‐filling duration, and grain quality (Sihag et al., 2024; Singh et al., 2023; Tanin et al., 2022). For instance, exposing wheat to high temperature at the anthesis stage linearly reduced the floret fertility from 85% at 24°C to 0% (i.e., complete abortion of pollen grains) at 35°C (Prasad & Djanaguiraman, 2014). Studies have also reported that every 1°C increase in temperature during anthesis and grain filling reduces grain yield by 6% (Zhao et al., 2017) and harvest index by 5% (Lawlor & Mitchell, 2000) in wheat. Additionally, heat stress reduces endosperm counts in the developing grains, thereby reducing the grain size and 1000‐grain weight, which results in reduced grain quality (Girousse, 2023).

Heat stress threatens global food security by reducing wheat production in major wheat‐growing areas of the world. Therefore, it is necessary to understand the genetic basis of heat stress tolerance as well as to develop heat‐resilient wheat cultivars. A first step toward achieving this is to tap into native genetic and phenotypic diversity to identify candidate genomic regions associated with heat stress tolerance. Several genome‐wide association studies (GWAS) and/or quantitative trait loci (QTLs) studies have been conducted in wheat to identify significant marker‐trait associations (MTAs) or QTLs associated with various agronomic, physiological, and biochemical traits under heat stress (Devate et al., 2022; Gudi et al., 2022; Khan et al., 2022; Maulana et al., 2018; Muhammad et al., 2020; Qaseem et al., 2019; Tanin et al., 2023; Tian et al., 2023). Most of these studies were conducted under terminal heat stress or at the grain‐filling stage with only few studies focusing on seedling heat stress tolerance (Fu et al., 2024; Khan et al., 2022; Maulana et al., 2018). Despite these efforts, the genetic mechanism(s) governing heat stress tolerance is not thoroughly understood. Moreover, most of these studies failed to identify the beneficial alleles and to develop the functional markers that can be utilized to develop heat‐resilient wheat cultivars. Therefore, it is necessary to identify the source of seedling heat stress‐tolerant lines and their genomic regions putatively involved in improved heat stress tolerance. To accomplish this, we evaluated a diverse panel of hexaploid spring wheat accessions, including landraces and cultivars, under heat stress and identified extremely heat‐tolerant lines. Moreover, a comprehensive GWAS, candidate gene (CG) mining underneath significant MTAs, and expression analysis helped us to identify the potential CGs involved in improved seedling heat stress tolerance.

## 2 MATERIALS AND METHODS

### 2.1 Plant materials

The experimental material consisted of 216 hexaploid wheat accessions with spring type growth habit (Table S1). These germplasm lines were selected from the exome‐sequenced wheat panel, which consisted of diverse accessions of tetraploid and hexaploid wheat lines belonging to landraces and cultivars (He et al., 2019). Seed materials were sourced from the United States Department of Agriculture‐National Small Grains Collection gene bank and grown for one round of purification and seed increase. Genotypic data were accessed from the online repository (http://wheatgenomics.plantpath.ksu.edu/1000EC).

### 2.2 Evaluation for seedling heat stress tolerance

The experiment was conducted using a two‐factor randomized complete block design with three replications. Seeds from each genotype were surface‐sterilized using 10% Clorox (for 20 min) and 70% ethanol (for 5 min) followed by rinsing three times with deionized water (diH_2_O). Disinfected seeds were kept in refrigerator (4°C) for 48 h to ensure uniform seed germination. Seeds from each genotype were sown in six cones (30 cm length and 5‐cm diameter) filled with autoclaved vermiculite. Three of the six cones representing three replications were placed in a growth chamber adjusted to 23°C (i.e., non‐heat stress treatment; T1), and the remaining three cones were placed in a separate growth chamber adjusted to 36°C (i.e., heat stress treatment; T2). Once the seeds germinated, growth chambers were adjusted to a 16‐h photoperiod (i.e., 16 h of light and 8 h of dark) for 12 days. On the 13th day, data on seedling characteristics such as shoot length (SL; cm), root length (RL; cm), root number (RN), coleoptile length (CL; cm), shoot fresh weight (SFW; mg), and root fresh weight (RFW; mg) were recorded. Precautions were taken to avoid water stress by providing sufficient amounts of water (via water containers) at 23°C for the non‐heat stress treatment (T1) and 36°C for the heat stress treatment (T2). Furthermore, containers were replaced with fresh water after every third day to avoid fungal growth.

**Core Ideas**

- Evaluating global spring wheat diversity panel identified highly heat‐tolerant lines.
- A genome‐wide association studies (GWAS) identified 23 marker‐trait associations (MTAs) associated with different seedling traits in wheat.
- Expression analysis pinpointed candidate genes (CGs) putatively involved in heat stress tolerance.

### 2.3 Phenotypic data analysis

Phenotypic data collected on seedling traits under non‐heat stress (T1) and heat stress (T2) treatments were subjected to mixed linear model (MLM) using META‐R. The META‐R relies on LME4 R‐package (Alvarado et al., 2020) to calculate the genotypic best linear unbiased estimates (BLUEs) for individual treatments. BLUE values were estimated by using the following model:

$$Y_{ij}=\mu +G_{i}+R_{j}+e_{ij}$$

where, *Y_ij_* is the trait of interest, *μ* is the grand mean, *G_i_* is the effect of *i*th genotype, *R_j_* is the effect of *j*th replication, *e_ij_* is the error associated with *i*th genotype and *j*th replication, which is assumed to be normally and independently distributed, with mean zero and homogeneity of variances as *σ* ^2^.

The broad‐sense heritability (h^2^_bs_) for seedling traits was calculated by using the following formula in META‐R:

$${h_{\mathrm{bs}}}^{2}=\frac{{\sigma_{g}}^{2}}{{\sigma_{g}}^{2}+{\sigma_{e}}^{2}/R}$$

where, *h*_bs_^2^ is the broad‐sense heritability, *σ*_g_^2^ is the genetic variance, *σ*_e_^2^ error variance, *R* is the number of replications.

BLUE values from heat stress treatment were divided from those from non‐heat stress treatments for each genotype to check the impact of heat stress. Moreover, this ratio was used to select extremely tolerant and sensitive genotypes for heat stress. We used paired *t*‐test to check the significant difference for each genotype under non‐heat stress and heat stress.

Pearson's correlation coefficient analysis among the seedling traits was assessed based on BLUEs using the “Corrplot” package built in the RStudio (R Core Team, 2018).

### 2.4 Single nucleotide polymorphisms (SNPs) genotyping and population statistics

The wheat accessions used in the present investigation were previously genotyped by capturing the exonic regions using the NimbleGen SeqCap EZ Reagent Kit Plus v2 (Roche) and sequenced on an Illumina HiSeq2000 instrument (He et al., 2019). Variant calling file containing a filtered set of single nucleotide polymorphisms (SNPs) (i.e., 3,573,809) was used to extract the genotypic information for the selected genotypes. Obtained genotypic information was subjected to data filtering using “TASSEL v5.2.84” to remove the SNPs with minor allele frequency of <5%, missing data of >20%, and heterozygosity of >20%. Beagle v4.1 was used to assign the missing genotypes from selected SNPs (Browning & Browning, 2007). Finally, the imputed SNPs (i.e., 302,524) were used for genome‐wide association analysis.

Population stratification was analyzed using Bayesian model‐based clustering, unsupervised principal component analysis (PCA), and kinship analysis. “STRUCTURE v.2.3.4” software was used for Bayesian model‐based clustering. In brief, structure analysis was done by setting the number of fixed sub‐populations to 10 (i.e., *k* = 10) with 10,000 MCMC (Markov chain Monte Carlo) replications and 10,000 burn‐in iterations (Pritchard et al., 2000). Then the web‐based program “STRUCTURE HARVESTER v0.6.9450” was used to determine the most likely number of sub‐populations by plotting ∆K and number of sub‐populations (https://taylor0.biology.ucla.edu/structureHarvester/). The unsupervised PCA was performed in “PLINK,” and the scatter plot showing grouping of genotypes was generated using R‐package. Kinship analysis was carried out to see the familial relationship among the genotypes using “GAPIT” through pairwise SNP comparison. Kinship plot depicting the genetic relatedness among genotypes was generated from square symmetric matrix.

Short‐range linkage disequilibrium (LD) was assessed by calculating squared allelic correlation (*r* ^2^) between SNP pairs within a 5000 Kb distance by utilizing “PopLDdecay software.” The LD‐decay plot was generated by (i) plotting estimated *r* ^2^ values against the genetic distance (in Kb) between SNPs and (ii) calculating the LD decay by fitting smooth locally weighted polynomial regression (LOESS) curve. Point at which the critical *r* ^2^ value of 0.2 intersects with the LOESS curve was identified as the LD decay point. LD decay analysis was conducted at the sub‐genomes and across the whole genome.

### 2.5 Association analysis and CG mining

Genome‐wide association analysis was carried out by utilizing the filtered set of SNPs (302,524) to identify significant MTAs. Analysis was performed using the general‐linear model (GLM), MLM, fixed and random model circulating probability unification (FarmCPU), and Bayesian information and LD iteratively nested keyway (BLINK) built in the R‐environment, “GAPIT v 3.0” (Genome association and prediction integrated tool) (Wang & Zhang, 2021). GLM and MLM control the false positives by incorporating the cofactors such as population structure (*Q*) in GLM and population structure (*Q*) and kinship (*K*) in the MLM. FarmCPU is a multi‐locus model that reduces false negatives by eliminating the confounding effects generated between test markers and the covariates (X. Liu et al., 2016). To avoid the overfitting of testing markers, FarmCPU considers associated markers as the fixed effects in the fixed effect model and was optimized in a maximum likelihood method in the MLM. BLINK improves the statistical power by employing LD and uses the Bayesian information content in a fixed‐effect model to overcome the computational load. Comparing all four models using quantile–quantile plots (Q–Q plots) suggested that FarmCPU performed better in fixing false positive and false negative associations. Therefore, FarmCPU was employed to identify significant MTAs. The Bonferroni correction's threshold for declaring significant MTAs is typically too stringent as it considers all SNPs in the dataset rather than independent tests. Therefore, we used the less stringent, arbitrary threshold of −log_10_(*p*) ≥ 5.0 to consider significant MTAs as suggested by Halder et al. (2023).

CG models within a 1 Mb region (500 Kb on either side) of each MTA were extracted using the BioMart tool present in Ensembl Plants (https://plants.ensembl.org/index.html). The 1 Mb window size for CG analysis was decided based on the results of LD decay analysis, which indicated an average whole genome LD block size of 1.5 Mb. Moreover, previous studies also suggested using a 1 Mb genomic region around significant MTA or QTL for CG analysis (Halder et al., 2023; Halladakeri et al., 2023). Functional descriptions of the identified CG models were obtained from the InterPro database (https://www.ebi.ac.uk/interpro/). The extracted CGs were subjected to in silico expression analysis using the wheat expression browser (i.e., ExpVIP) (http://www.wheat‐expression.com/). For this purpose, the expression dataset involving the transcriptomic analysis of seedling heat stress (1 and 6 h) in the heat‐tolerant wheat variety TAM‐107 was used (Z. Liu et al., 2015). Finally, the CGs with at least two‐fold change in the expression value were considered as differentially expressed genes (DEGs) as suggested in Gudi et al. (2022).

## 3 RESULTS

### 3.1 Phenotypic evaluation of wheat seedlings for heat stress tolerance

**FIGURE 1:**
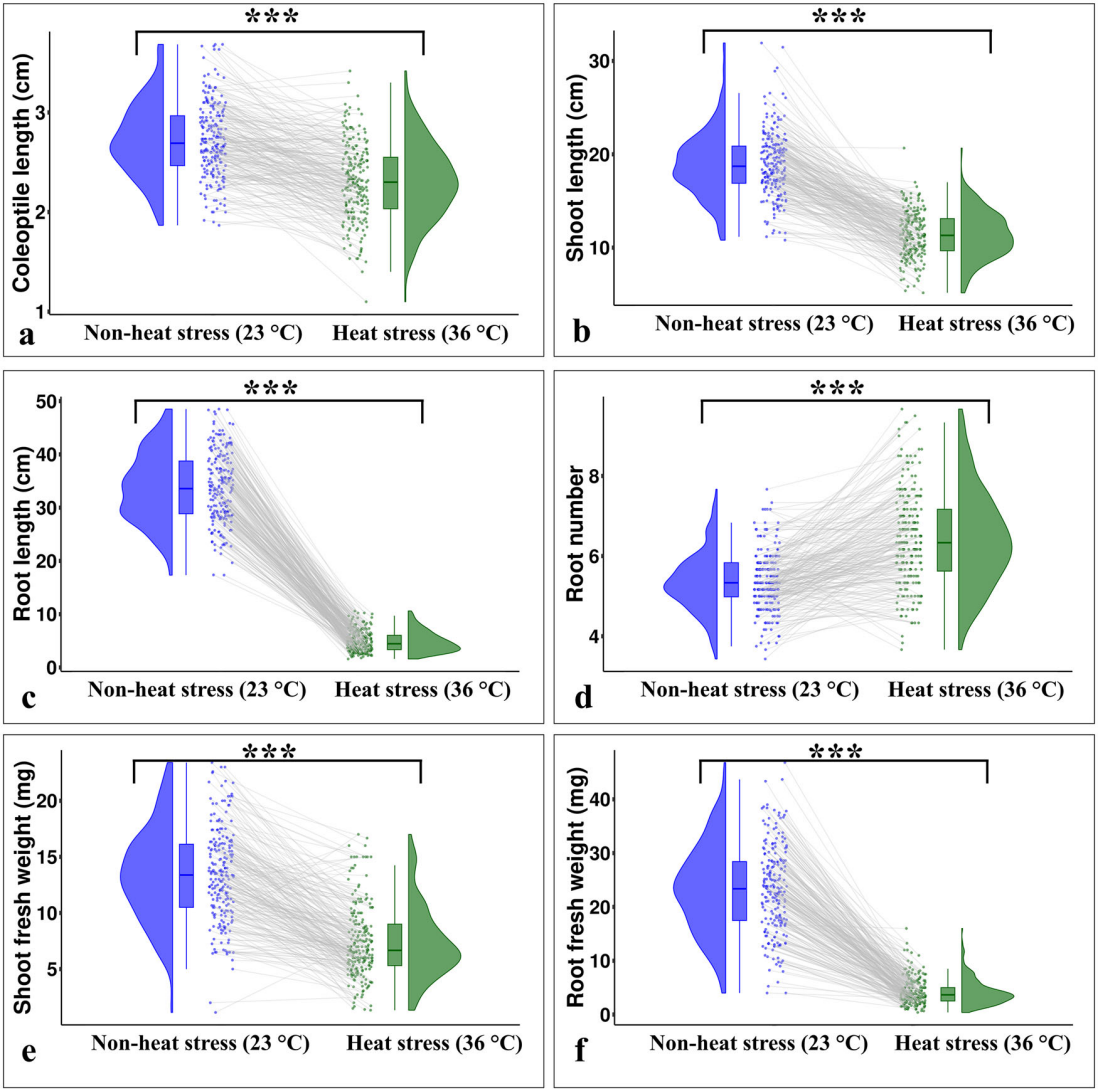
Phenotypic variation for seedling traits under non‐heat stress (23°C) (blue) and heat stress (36°C) (green) treatment: (a) coleoptile length (cm); (b) shoot length (cm); (c) root length (cm); (d) root number; (e) shoot fresh weight (mg); (f) root fresh weight (mg). In each treatment, the kernel density plot represents the data distribution; box plot represents the minimum, first quartile, mean/median, third quartile, and maximum values in the dataset; and dots represent the individual genotypes. The gray lines depict the performance of same genotype in non‐heat stress and heat stress treatments with connecting dots. ***Significant at *p* < 0.001.

**FIGURE 2:**
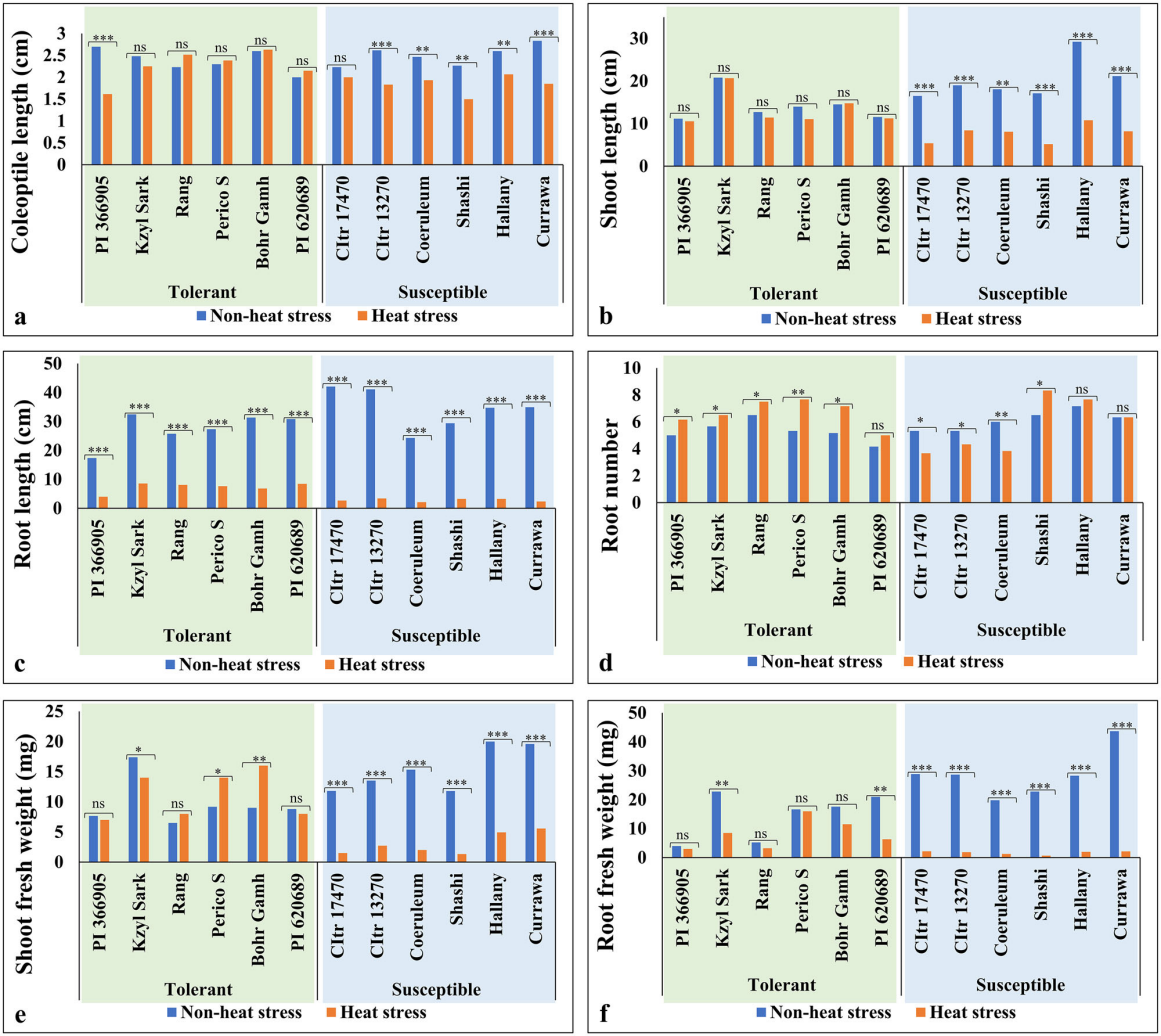
Comparing the performance of six highly tolerant (light green box) and six highly sensitive (light blue box) lines under non‐heat stress (23°C) (blue bars) and heat stress (36°C) (orange bars) treatments for (a) coleoptile length (cm); (b) shoot length (cm); (c) root length (cm); (d) root number; (e) shoot fresh weight (mg); (f) root fresh weight (mg). A paired *t*‐test was used to check the level of significance between non‐heat stress (23°C) and heat stress (36°C) treatment for each genotype (level of significance: * < 0.05%; ** < 0.01%; *** < 0.001; ns, non‐significance).

Hexaploid wheat accessions selected from the exome‐sequenced panel were assessed for seedling heat stress tolerance by measuring multiple seedling traits (SL, RL, CL, RN, SFW, and RFW) under heat stress (36°C) and non‐heat stress (23°C) treatments. The BLUE values estimated from the phenotypic data showed significant variations for all seedling traits under non‐heat stress (23°C) and heat stress (36°C) treatments (Table 1; Figure 1). Higher phenotypic variation was observed under non‐heat stress (23°C) treatment than under heat stress (36°C) for most of the seedling traits (except CL and RN). Heat stress negatively affected all seedling traits with the maximum effect on RL (85.6% reduction) and the minimum effect on CL (15.44% reduction). Furthermore, a significant increase in RN (20%) was observed under heat stress compared to the non‐heat stress treatment. To check the stability of genotype performance under heat stress, we compared the top 25% (i.e., 54 genotypes) ranked under non‐heat stress with their ranking under heat stress for each trait. Among these top performers, only 22 genotypes maintained their top 25% ranking under heat stress for CL, 15 for SL, 16 for RL, 22 for RN, 24 for SFW, and 19 for RFW (Figure 1). The remaining genotypes failed to maintain their ranking under heat stress, indicating a reduction in performance for these genotypes. Moreover, based on performance of genotypes under heat stress, we identified six highly tolerant (i.e., PI 366905, Kzyl Sark, Rang, Perico S, Bohr Gamh, and PI 620689) and six highly susceptible (i.e., CItr 17470, CItr 13270, Coeruleum, Shashi, Hallany, and Currawa) genotypes (Figure 2).

**TABLE 1:** Summary statistics for the seedling traits under non‐heat stress (23°C) and heat stress (36°C) treatments.

Statistic	CL	SL	RL	RN	SFW	RFW
**Non‐heat stress (23°C)**						
Mean ± LSD	2.72 ± 0.5***	18.92 ± 4***	33.76 ± 6.05***	5.35 ± 1.2***	13.42 ± 4.94***	23.23 ± 4.24***
Range	1.87–3.68	10.8–31.92	17.33–48.5	3.43–7.67	1.14–23.4	4–46.82
CV (%)	11.55	13.17	11.17	13.94	22.96	11.39
Heritability	0.77	0.83	0.89	0.66	0.82	0.96
**Heat stress (36°C)**						
Grand mean	2.3 ± 0.46***	11.38 ± 2.81***	4.86 ± 1.49***	6.42 ± 1.8***	7.38 ± 3.04***	4.18 ± 2.64***
Range	1.1–3.42	5.17–20.67	1.6–10.6	3.67–9.67	1.33–17	0.4–16
CV (%)	12.39	15.37	19.15	17.51	25.69	39.42
Heritability	0.82	0.83	0.93	0.71	0.88	0.85
**Percent reduction**	15.44	39.85	85.6	−20	45.01	82.01

Abbreviations: CL, coleoptile length (cm); CV, coefficient of variance; LSD, least significant difference; RFW: root fresh weight (mg); RN, root number; RL, root length (cm); SFW, shoot fresh weight (mg); SL, shoot length (cm).

***Significant at *p* < 0.001.

**FIGURE 3:**
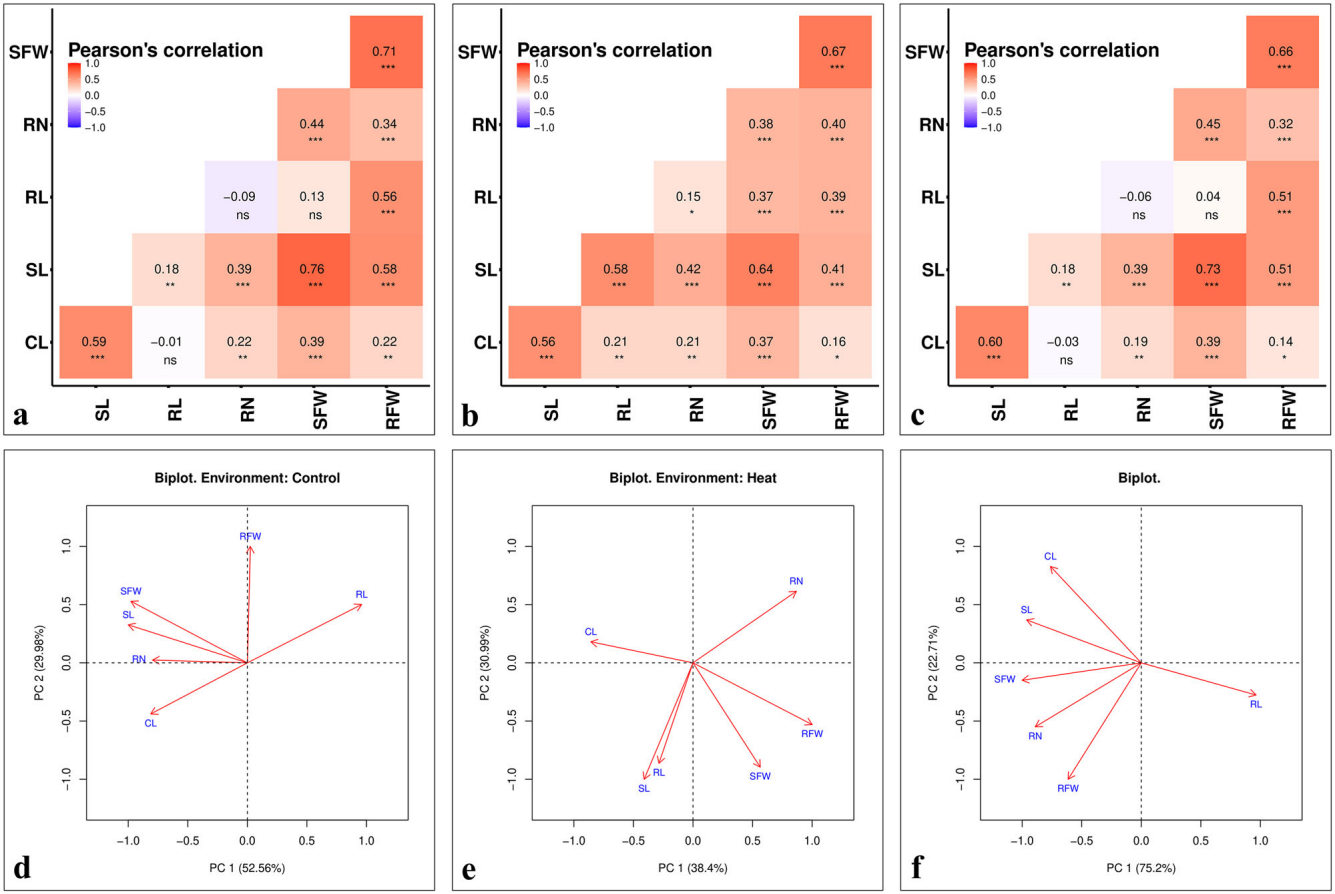
Pearson's correlation coefficient among the seedling traits under non‐heat stress (23°C) (a), heat stress (36°C) (b), and combined dataset from non‐heat stress and heat stress treatments (c); and the principal component analysis (PCA) analysis for seedling traits under non‐heat stress (d), heat stress (e), and combined dataset from non‐heat stress (23°C) and heat stress (36°C) treatments (f). CL, coleoptile length (cm); RFW, root fresh weight (mg); RN, root number; RL, root length (cm); SFW, shoot fresh weight (mg); SL, shoot length (cm). *Significant at *p* < 0.05; **Significant at *p* < 0.01; ***Significant at *p* < 0.001.

The h^2^_bs_ estimated using BLUE values revealed the different genetic architecture of seedling traits under non‐heat stress and heat stress treatments (Table 1). The maximum and minimum heritability was observed for RFW (96%) and RN (66%), respectively, under the non‐heat stress treatment. However, under heat stress, the maximum heritability was observed for RL (93%) and the minimum heritability was observed for RN (71%). Furthermore, the heritability estimates were higher for all seedling traits (except RFW) under heat stress compared to the non‐heat stress treatment. Pearson's correlation coefficient analysis revealed a significant positive association (*p*‐value < 0.05) among seedling traits under non‐heat stress and heat stress treatments as well as for the combined dataset except for RL with CL, RN, and SFW under non‐heat stress and combined dataset (Figure 3a–c). Correlation coefficient analysis revealed a notable shift in the trait relationship under non‐heat stress and heat stress treatments. Most prominent changes were observed for SL versus RL and SFW versus RL (Figure 3a,b). Under non‐heat stress, SL and RL showed a weak association (*r* = 0.18; *p*‐value < 0.01), whereas this relationship was strengthened under heat stress treatment (*r* = 0.58; *p*‐value < 0.001). Likewise, RL was not correlated with SFW under non‐heat stress, but showed a strong positive correlation under heat stress (*r* = 0.18; *p*‐value < 0.01). These results suggests that genotypes maintaining long RL will support the better shoot growth and biomass accumulation under heat stress. Correlation results were further confirmed by PCA biplots, which showed higher levels of association among the seedling traits (Figure 3d–f).

### 3.2 Genotypic information, population structure, and LD analysis

Of the total 7.3 million SNPs, 302,524 filtered SNPs were used for the GWAS. The chromosome with the most SNPs was chromosome 2B (i.e., 26,385), whereas chromosome 4D had the least (i.e., 919) (Table S2). Among the sub‐genomes, the B‐genome had the highest number of SNPs (i.e., 148,165) and the D‐genome had the lowest number of SNPs (i.e., 27,052) (Table S2).

**FIGURE 4:**
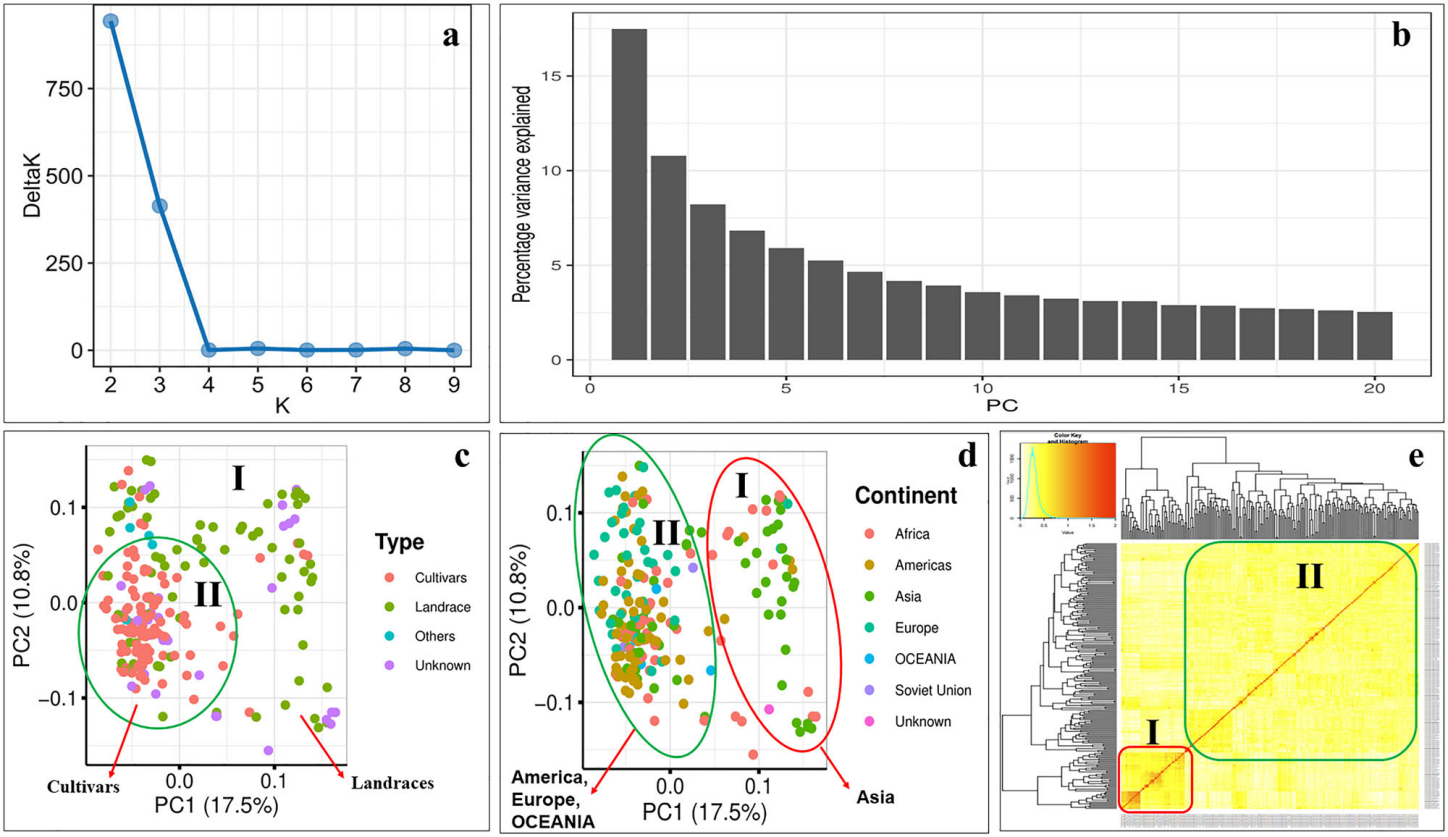
Population structure analysis of the diverse hexaploid spring wheat collection: (a) Evanno plot of Delta‐K statistic obtained from Bayesian model‐based clustering in the STRUCTURE software; (b) scree plot for first 20 principal components obtained from principal component analysis (PCA); (c) PCA‐based scatter plot showing the grouping of wheat accessions based on type of germplasm lines; (d) PCA‐based scatter plot showing the grouping of wheat accessions based on origin; (e) kinship plot showing the familial relationship among the wheat germplasm lines.

Bayesian model‐based clustering and PCA revealed two subgroups in the association panel (Figure 4a,b). The first two PCs explained 28.3% (i.e., 17.5% by PC1 and 10.8% by PC2) of the total genetic variation. These PCs were used to differentiate the genotypes based on type (i.e., cultivated, landraces, and unknown) and origin (i.e., continent wise) (Figure 4c). Group I consisted mainly of landraces collected from Asia and Africa with a few genotypes collected from Europe and the Americas (Figure 4d). However, Group II included both cultivated and landraces collected from Europe and the Americas with a few genotypes from Asia and Africa. These results were further confirmed by kinship (*K*) analysis using “GAPIT,” which revealed two subgroups in the association panel (Figure 4e). The LD‐decay analysis using critical *r* ^2^ value identified the LD block size of 1.5 Mb at the whole genome level (Figure S1). However, the LD‐decay analysis showed a differential pattern across the three sub‐genomes; that is, with the A (∼1.2 Mb) and B (∼1.3 Mb) sub‐genomes showed smaller LD block sizes compared to that of the D sub‐genome (i.e., >9 Mb) (Figure S1).

### 3.3 Genome‐wide association analysis

**FIGURE 5:**
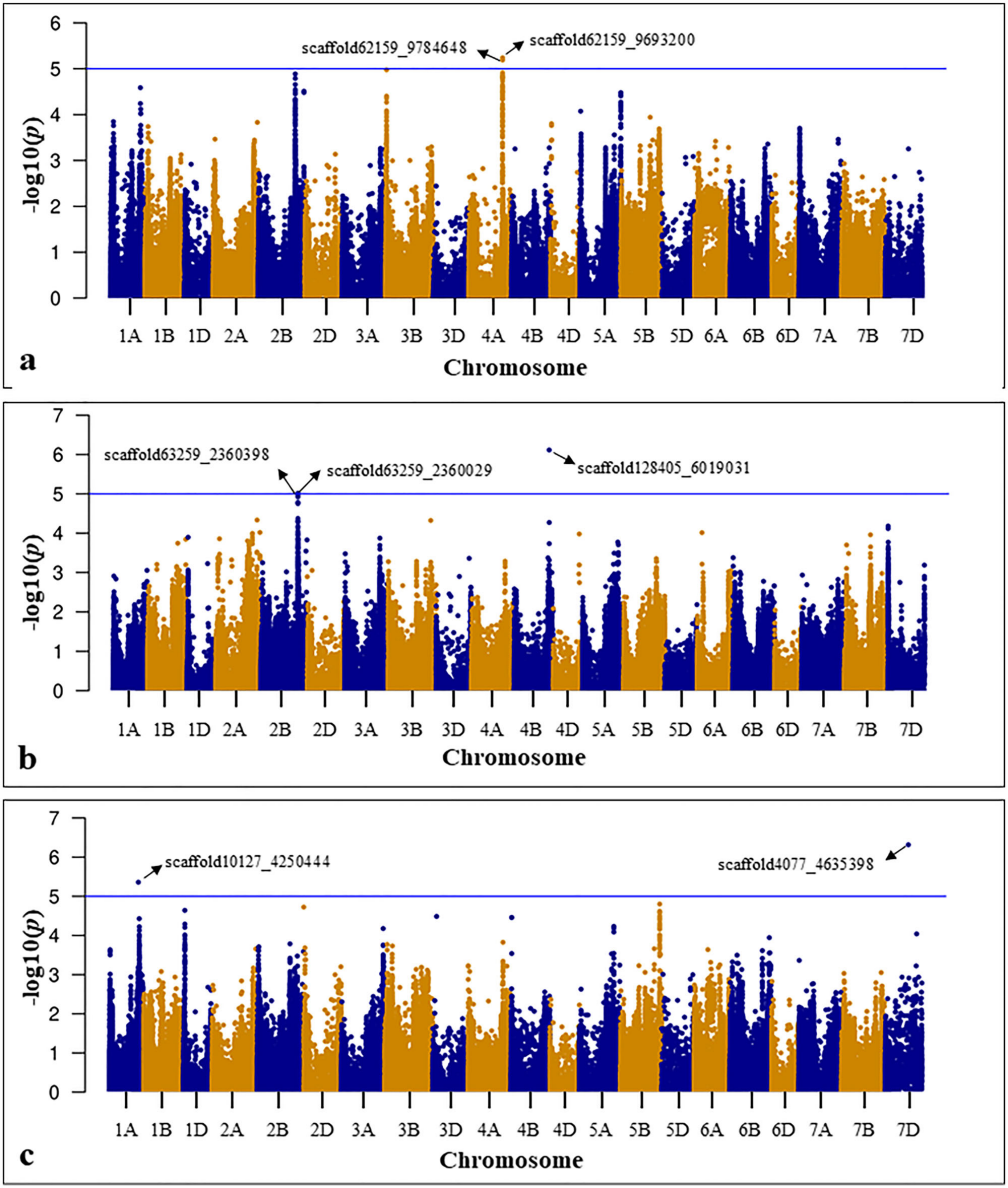
Marker‐trait associations (MTAs) identified for seedling traits under non‐heat stress (23°C) treatment: (a) MTAs associated with shoot length (SL; cm); (b) MTAs associated with root number (RN); (c) MTAs associated with root fresh weight (RFW; mg).

**FIGURE 6:**
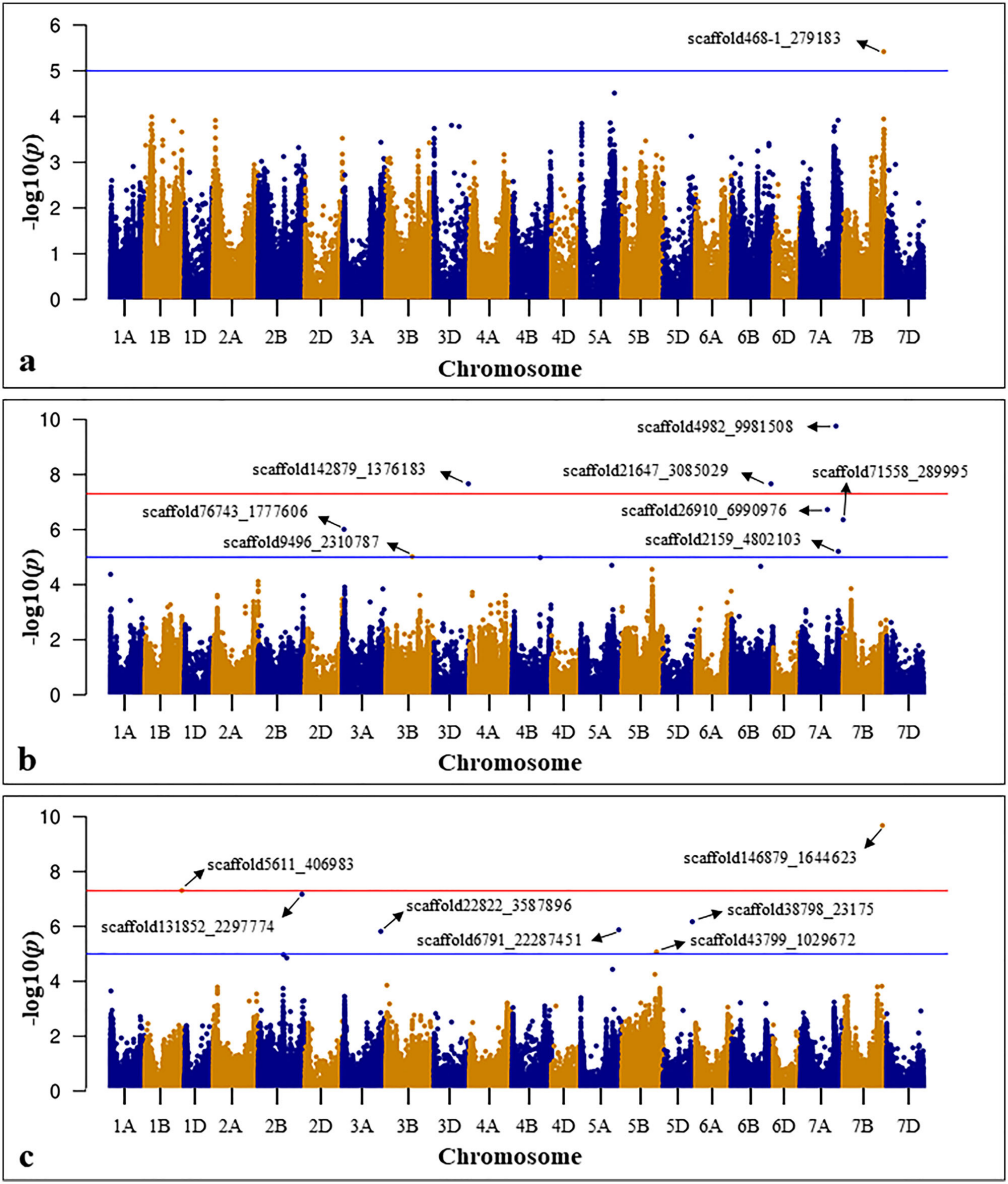
Marker‐trait associations (MTAs) identified for seedling traits under heat stress (36°C) treatment: (a) MTAs associated with shoot fresh weight (SFW; mg); (b) MTAs associated with root length (RL; cm); (c) MTAs associated with root fresh weight (RFW; mg).

**FIGURE 7:**
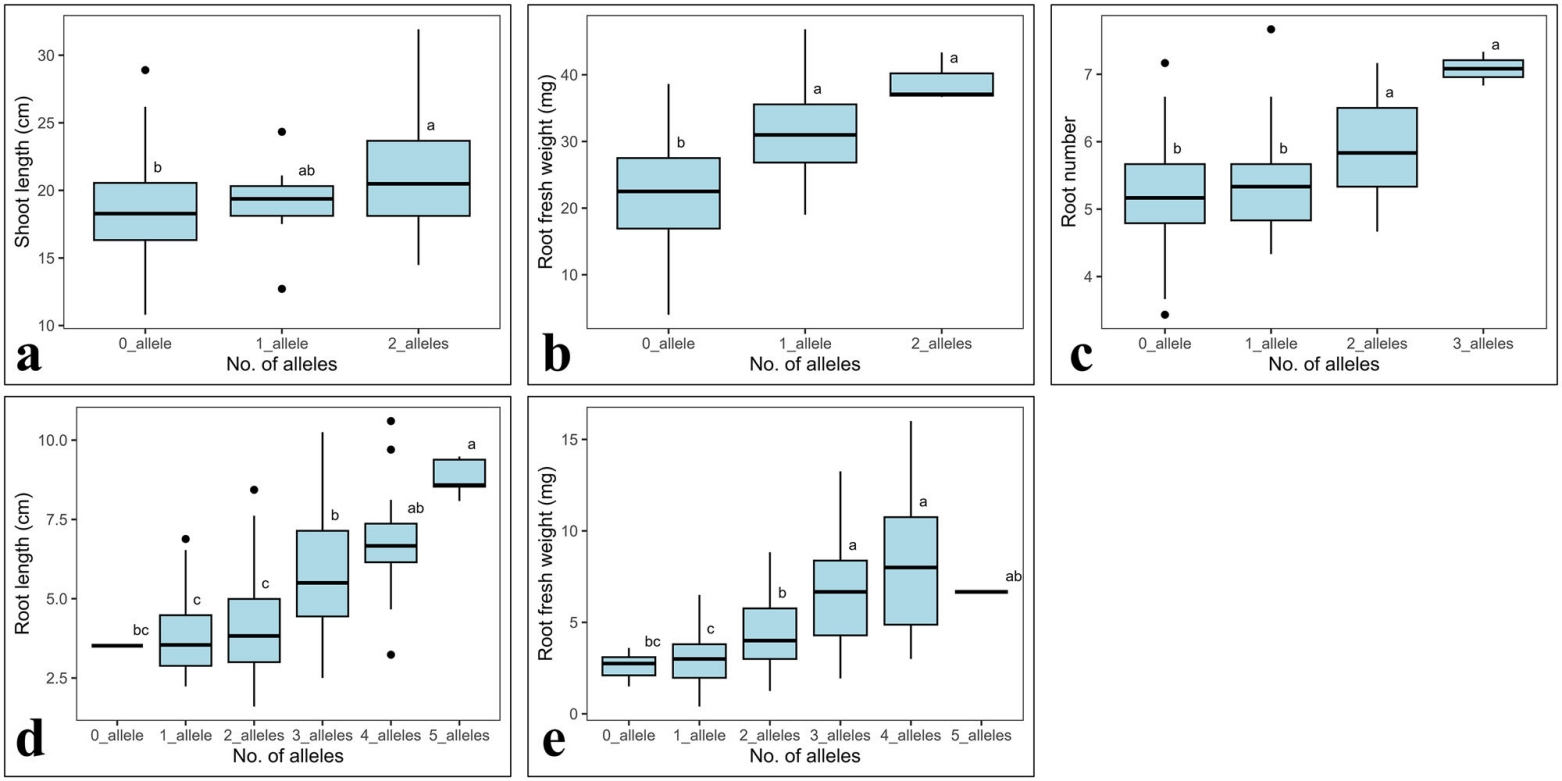
Additive effect of favorable alleles on: (a) shoot length (SL; cm) under non‐heat stress (23°C) treatment; (b) root fresh weight (RFW; mg) under non‐heat stress (23°C) treatment; (c) root number under non‐heat stress (23°C) treatment; (d) root length (RL; cm) under heat stress (36°C) treatment; (e) root fresh weight (RFW; mg) under heat stress (36°C) treatment. Letters (a, b, c) in the boxplots represent the statistical significance difference (at 5%) between group means based on Tukey's HSD test.

The BLUE values obtained from phenotypic data on seedling traits were subjected to GWAS using GLM, MLM, FarmCPU, and BLINK. However, comparing GWAS results using Q–Q plots showed better results for FarmCPU, and hence we proceed with FarmCPU. GWAS identified 23 significant MTAs associated with different seedling traits, of which 21 are unique MTAs. Moreover, among 23 MTAs, seven were identified under non‐heat stress and 16 under heat stress treatment (Table 1; Figure S2 and S3). Of the seven MTAs identified under non‐heat stress treatment, two were associated with SL, three were associated with RN, and two were associated with RFW (Figure 5). MTAs associated with RFW had the highest SNP effect of 4.68 (scaffold10127_4250444) and 6.19 (scaffold4077_4635398). Of the 16 MTAs identified under heat stress treatment, one was associated with SFW, eight were associated with RL, and seven were associated with RFW (Figure 6). Four of these MTAs, scaffold468‐1_279183, scaffold5611_406983, scaffold38798_23175, and scaffold146879_1644623, can be considered as most important MTAs, as they possess high allelic effect of >1 (Table 2). Interestingly, a heat‐tolerant line, Bohr Gamh (PI 462106), collected from middle Asia possesses positive alleles for all these MTAs. Similarly, tolerant lines, PI 366905, have positive alleles for three MTAs (scaffold468‐1_279183, scaffold5611_406983, and scaffold38798_23175), Kzyl Sark has positive alleles for scaffold468‐1_279183, and Perico S has positive alleles for scaffold5611_406983. However, two tolerant lines, Rang and PI 620689, did not have positive alleles for these four MTAs, suggesting that there might be other genomic regions giving tolerance in these genotypes. Interestingly, it was noticed that all six sensitive genotypes possess negative alleles for these four MTAs, further supporting the possible involvement of these MTAs in heat stress tolerance. Furthermore, allelic effect estimation revealed that the favorable alleles of MTAs identified under non‐heat stress improved seedling performance under non‐heat stress only (Figure S4a–f), except for MTA, scaffold4077_4635398, which improved seedling performance under both non‐heat stress and heat stress treatments (Figure S4g). Similarly, of the 16 MTAs identified under heat stress, favorable alleles for 12 MTAs improved seedling performance under heat stress only (Figure S4), whereas the favorable alleles of scaffold468‐1_279183 (Figure S4h), scaffold21647_3085029 (Figure S4l), scaffold2159_4802103 (Figure S4p), and scaffold22822_3587896 (Figure S4s) improved seedling performance under both non‐heat stress and heat stress treatments. Therefore, only 12 MTAs identified under heat stress whose favorable alleles did not change the performance across treatments are useful in breeding for heat tolerance. Furthermore, we estimated the additive effect of favorable alleles on the phenotypic performance, which indicated that the genotypes with more favorable alleles had higher performance under heat stress treatment (Figure 7).

**TABLE 2:** List of marker‐trait associations (MTAs) identified for seedling traits under non‐heat stress (23°C) and heat stress (36°C) treatments.

Trait	MTAs/SNPs	Chromosome	Physical position (bp)	Allelic effect	LOD score
**Non‐heat stress (23°C)**					
SL	scaffold62159_9784648	4A	586339854	1.46	5.19
	scaffold62159_9693200	4A	586431302	−1.46	5.24
RN	scaffold63259_2360029	2B	639219929	−0.35	5.01
	scaffold63259_2360398	2B	639220298	0.27	5
	scaffold128405_6019031	4B	603166442	0.6	6.12
RFW	scaffold10127_4250444	1A	499798735	4.68	5.36
	scaffold4077_4635398	7D	404242254	6.19	6.32
**Heat stress (36°C)**					
SFW	scaffold468‐1_279183	7B	701158105	−1.43	5.42
RL	scaffold76743_1777606	3A	26518037	−0.6	6.01
	scaffold9496_2310787	3B	454645221	0.4	5.02
	scaffold142879_1376183	3D	595273895	0.65	7.67
	scaffold21647_3085029	6B	683822994	−0.66	7.66
	scaffold26910_6990976	7A	464572920	0.79	6.72
	scaffold4982_9981508	7A	612703273	0.97	9.76
	scaffold2159_4802103	7A	652687632	0.77	5.21
	scaffold71558_289995	7A	735776454	0.66	6.36
RFW	scaffold5611_406983	1B	638934103	1.15	7.31
	scaffold131852_2297774	2B	751183272	−0.89	7.17
	scaffold22822_3587896	3A	661835680	0.78	5.82
	scaffold6791_22287451	5A	657547002	−0.97	5.88
	scaffold43799_1029672	5B	599554479	0.5	5.07
	scaffold38798_23175	5D	506095232	−1.47	6.17
	scaffold146879_1644623	7B	678622161	−1.29	9.68

Abbreviations: LOD, logarithm of odds; MTAs, marker‐trait associations; RFW, root fresh weight (mg); RN, root number; RL, root length (cm); SFW, shoot fresh weight (mg); SNPs, single nucleotide polymorphism; SL, shoot length (cm).

### 3.4 CG and expression analysis

CG analysis from the genomic regions spanning 21 significant MTAs identified 310 unique gene models (Table S3). The highest number of CGs (27) was identified in scaffold6791_22287451, located on chromosome 5A, whereas the lowest number of CGs (4) was identified in scaffold26910_6990976, located on chromosome 7A. Of the 310 unique gene models, 263 had functional annotation, whereas the remaining 47 genes were uncharacterized. Multiple gene models with similar gene functions were frequently detected from the genomic regions involving different MTAs. For instance, 19 gene models harbored protein kinase (PK) domains, eight had ubiquitin‐like domains, six had zinc finger and RNA recognition motif domains, five had NB‐ARC and EF‐hand domains, four were similar to cytochrome P450 proteins, three had F‐box domains, and others are listed in Table S3.

**FIGURE 8:**
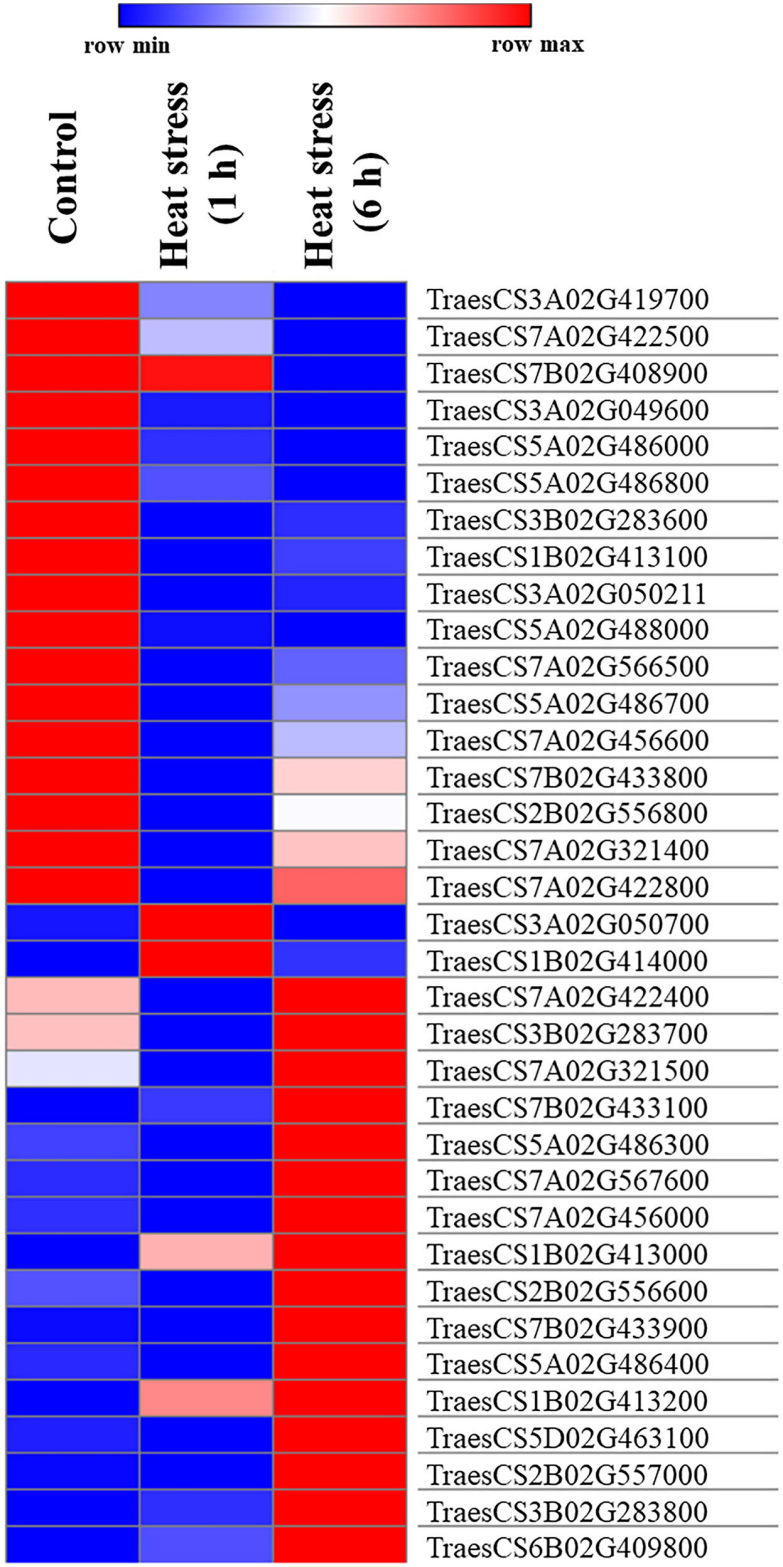
Heat map showing the differentially expressed genes (DEGs) under non‐heat stress (23°C) and heat stress (36°C) treatment (1 and 6 h).

CG models (i.e., 310) extracted from genomic region spanning MTAs were subjected to in silico expression analysis using transcriptomic dataset involving seedling heat stress (1 and 6 h) and identified total of 35 DEGs (Z. Liu et al., 2015) (Figure 8; Table S4). Among the 35 DEGs, 15 were down‐regulated and 20 were up‐regulated under heat stress treatment. Of the 15 down‐regulated genes, the expression of two genes was reduced after 1 h of heat stress, 12 genes were reduced after 6 h of heat stress, and one gene was reduced after both 1 and 6 h of heat stress. Similarly, of the 20 up‐regulated genes, the expression of 10 genes increased after 1 h of heat stress, two genes increased after 6 h of heat stress, and eight genes expressed after both 1 and 6 h of heat stress.

Based on the gene functional description and the information available from the published literature, 13 of the 35 DEGs were found to be involved in heat stress tolerance, and these genes were considered as high‐confidence CGs (Table 3). These CGs were predicted to encode proteins belonging to the following classes: PK, fatty acid hydroxylase, pectinacetylesterase, UDP‐glucuronosyl/UDP‐glucosyltransferase, pyrophosphate‐energized proton pump, EF‐hand, basic‐leucine zipper (bZIP), DNA methylase, N‐6 adenine‐specific conserved site, and thioredoxin.

**TABLE 3:** Differentially expressed high‐confidence candidate genes (CGs) associated with seedling heat stress tolerance.

Sl. No.	Trait	Gene ID	Gene position (bp)	GO term name	Interpro description
1	RL	TraesCS3A02G049600	26229850‐26234135	ATP binding	Protein kinase domain
2		TraesCS3A02G050211	26469173‐26482725	ATP binding	Protein kinase domain
3		TraesCS3B02G283600	454656825‐454657602	–	–
4		TraesCS3B02G283700	454668034‐454672347	–	–
5		TraesCS6B02G409800	684220072‐684221496	Protein‐disulfide reductase activity	Thioredoxin domain
6		TraesCS7A02G422500	612990989‐612994043	ATP binding	Protein kinase domain
7	SFW	TraesCS7B02G433100	700723840‐700724301	Calcium ion binding	EF‐hand domain
8		TraesCS7B02G433800	701185893‐701191043	Integral component of membrane	Pyrophosphate‐energised proton pump
9	RFW	TraesCS2B02G556600	751317909‐751324133	DNA‐binding transcription factor activity	Basic‐leucine zipper domain
10		TraesCS5A02G486000	657181008‐657184937	Hydrolase activity	Pectinacetylesterase/NOTUM
11		TraesCS5A02G486400	657205537‐657208108	Nucleic acid binding	DNA methylase, N‐6 adenine‐specific, conserved site
12		TraesCS5A02G486800	657394482‐657396507	Hexosyltransferase activity	UDP‐glucuronosyl/UDP‐glucosyltransferase
13		TraesCS7B02G408900	678124719‐678128411	Integral component of membrane	Fatty acid hydroxylase

Abbreviations: GO, gene ontology; RFW, root fresh weight; RL, root length; SFW, shoot fresh weight.

## 4 DISCUSSION

Heat stress significantly reduces grain yield and quality by affecting various developmental stages of wheat, including seedling and reproductive stages. Phenotypic screening at early developmental stages for seed germination and seedling vigor enables high‐throughput evaluation of a large number of germplasm lines under controlled condition. Moreover, seedling evaluation accelerates the breeding process by allowing early selection of promising genotypes. Although seedling performance observed under controlled environment does not always repeat during the adult stage or at field conditions (due to involvement of combination of stresses), several studies reported that the genotypes performing well under seedling heat stress also tend to exhibit improved tolerance at the adult stage with a strong positive correlation (*r*  =  0.6930) (Alsamadany, 2016; Collins et al., 2017; Lu et al., 2022). Studies also reported the collocated QTLs and/or heat induced genes for the seedling stage and reproductive stage (Li et al., 2012). Therefore, we studied the effect of heat stress on six seedling traits (SL, RL, CL, RN, SFW, and RFW) associated with seedling vigor on 216 diverse spring wheat accessions. We observed a huge genetic variation for studied traits under heat stress. On average, wheat accessions showed reduced performance under heat stress for SL, RL, CL, SFW, and RFW, whereas RN was substantially increased. Compared to optimum temperature, high‐temperature stress slows down seed imbibition and oxygen uptake and inhibits the mobilization of seed reserves from endosperm to embryo, thereby delays or reduces the seed germination (Essemine et al., 2010). Moreover, direct exposure of germinating seeds to high‐temperature stress affects the rate of development of radicle and coleoptile tissues, thereby reduces seedling vigor by reducing root and shoot traits, respectively. Similarly, we observed a negative effect of heat stress on shoot and root traits. Furthermore, we observed that the impact of heat stress was more on root traits (RL and RFW) compared to shoot traits (CL, SL, and SFW). This is owing to the direct exposure of root tissue to increased soil temperature, which disrupts the cell division and meristematic tissue growth, thereby reduce primary root growth. Moreover, heat stress affects roots indirectly by creating increased water and nutrient demand for shoots or by decreasing carbon supply from shoots to roots, thereby reduces root biomass. In contrast, shoots have some protection from temperature fluctuations due to their exposure to air and the ability to adjust leaf orientation or canopy temperature to reduce heat absorption.

Identifying contrasting germplasm lines is crucial for understanding the molecular and genetic mechanisms underlying heat stress tolerance in crops (Janni et al., 2020). Moreover, heat‐tolerant lines can serve as valuable starting material for developing heat‐resilient wheat cultivars. In this study, we observed less impact of heat stress on six germplasm lines (i.e., PI 366905, Kzyl Sark, Rang, Perico S, Bohr Gamh, and PI 620689). These lines can be utilized in future breeding program to develop the heat‐resilient wheat cultivars.

Correlation studies reveal the intrinsic relationship among different traits and also help in indirect selection for stress‐tolerant traits. In this study, we observed a strong positive correlation among shoot and root traits, indicating that heat‐induced damage to roots impairs their ability to absorb sufficient water and nutrients, thereby limiting photosynthesis and resulting in a proportional decrease in shoot growth.

Plants have developed a range of adaptive mechanisms to mitigate the effects of heat stress. Identifying genomic regions associated with improved heat stress tolerance and incorporating them into cultivars helps in developing stress‐resilient crops. In this study, we used GWAS to identify 23 MTAs associated with seedling traits on 15 chromosomes (Figures 5 and 6; Table 2). Additionally, by comparing our results with published results of Khan et al. (2022), we identified five overlapping QTLs/MTAs (i.e., scaffold10127_4250444, scaffold4077_4635398, scaffold21647_3085029, scaffold5611_406983, and scaffold38798_23175) for seedling traits. Significant MTAs identified through GWAS serve as potential targets for extracting putative CGs associated with targeted traits (Devate et al., 2022; Rathan et al., 2022).

Integrating favorable alleles from multiple QTLs in same genetic background is an effective breeding strategy to optimize plant traits for stress tolerance (Fan et al., 2019; You et al., 2021). In this study, we noticed the significant additive effect of favorable alleles for different seedling traits under non‐heat stress (for SL, RFW, and RN) (Figure 7a–c) and heat stress (for RL and RFW) (Figure 7d,e) treatments. Genotypes with >1 favorable allele improved the seeding performance more than those with no favorable alleles, which signifies the importance of combining multiple favorable alleles to improve heat stress tolerance.

A SNP located on chromosome 3A (scaffold76743_1777606) is linked with two putative genes (*TraesCS3A02G049600* and *TraesCS3A02G050211*) encoding PK domain. PKs, including receptor‐like kinases (RLKs), mitogen‐activated PKs (MAPKs) cascades, calcium‐dependent PKs (CDPKs/CPKs), and calcineurin B like interacting PKs, play important roles in stress sensing and signal transduction and help in responding to prevailing stresses. For instance, MAPKs regulate the plant's response to multiple abiotic stresses, including heat and drought stress (de Zelicourt et al., 2016), and RLKs such as guard cell hydrogen peroxide‐resistant 1 are necessary for closing stomata under drought stress by regulating the synthesis of abscisic acid and hydrogen peroxide (Ding et al., 2013). Similarly, CPKs such as *cpk28* regulate the phosphorylation of ascorbate peroxidase (POD) and thereby improve heat stress tolerance (Hu et al., 2021). SNP located on chromosome 7B (scaffold468‐1_279183) linked to a gene, *TraesCS7B02G4338000*, which encodes for the proton‐pumping pyrophosphatases. These pyrophosphatases act as a backup system for converting the energy stored in pyrophosphates into electrochemical gradients under various stresses. For instance, the overexpression of H^+^‐PPase *AVP1* (Arabidopsis vacuolar H+‐PPase) results in increased SUMOylation, which ultimately leads to improved plant growth and development under heat, cold, drought, salt, and nutrient stresses (Gaxiola et al., 2012; Park et al., 2005; Patir‐Nebioglu et al., 2019; Schilling et al., 2017). Similarly, we identified a gene (*TraesCS2B02G556600*) linked to scaffold131852_2297774 encoded to bZIP transcription factors (TFs). These TFs regulate the expression of genes involved in stress tolerance and increase the plant's adaptability to heat, drought, salt, and cold stresses by enhancing the activity of antioxidant enzymes such as catalase, POD, and superoxide dismutase (Yu et al., 2020). Similarly, genes encoding a thioredoxin domain‐containing protein, a fatty acid hydroxylase, UDP‐glucosyltransferase, pectinacetylesterase, and an EF‐hand domain‐containing proteins. These proteins are known to provide heat stress tolerance through calcium signaling and protein‐disulfide reductase activity (Dong et al., 2020; Patir‐Nebioglu et al., 2019). We also identified two high‐confidence DEGs (*TraesCS3B02G283600* and *TraesCS3B02G283700*) with unknown functions. These might represent new classes of genes that confer tolerance to heat stress. Future studies may lead to better functional characterization of these genes to identify the underlying mechanisms of stress tolerance. Moreover, the combination of favorable alleles for multiple MTAs identified in this study can be integrated into common genetic background to develop heat‐resilient wheat varieties.

## 5 CONCLUSION

In this study, we observed a negative impact of heat stress on seedling traits and also identified highly tolerant lines for heat stress. These lines can be utilized in breeding programs to develop resilient wheat varieties with sustainable yield under changing climatic conditions. Additionally, our study identified significant MTAs and putative CGs associated with heat stress tolerance. Heat‐tolerant lines and the putative genes identified in this study provide the foundation for developing functional markers and also help in marker‐assisted breeding of heat‐resilient wheat varieties. Moreover, these results provide the foundation for future studies focusing to understand the genetic basis of heat stress tolerance in wheat.

## AUTHOR CONTRIBUTIONS

**Santosh Gudi**: Conceptualization; data curation; formal analysis; methodology; project administration; software; visualization; writing—original draft. **Jatinder Singh**: Formal analysis; visualization; writing—original draft. **Harsimardeep Gill**: Formal analysis; visualization. **Sunish Sehgal**: Writing—review and editing. **Justin D. Faris**: Writing—review and editing. **Upinder Gill**: Conceptualization; investigation; supervision; writing—review and editing. **Rajeev Gupta**: Conceptualization; funding acquisition; investigation; project administration; resources; supervision; writing—review and editing.

## CONFLICT OF INTEREST STATEMENT

The authors declare no conflicts of interest.

## Supporting information




**Supplementary Table 1**. List of 216 germplasm lines used for association analysis in the present study.
**Supplementary Table 2**. Number of single nucleotide polymorphisms (SNPs) distributed along the chromosomes and the sub‐genomes, and the total number of SNPs in whole genome.
**Supplementary Table 3**. List of unique gene models identified from the significant marker‐trait associations (MTAs) associated with seedling traits under non‐heat stress (23°C) (blue) and heat stress (36°C) (green) treatment.
**Supplementary Table 4**. List of differentially expressed genes (DEGs) identified in the present study.

**Supplementary Figure 1**. Analysis of linkage disequilibrium (LD) at the whole genome and sub‐genome levels in the hexaploid spring wheat collection. **(a)** LD decay analysis of the whole genome; **(b)** LD decay analysis for the A‐sub‐genome; **(c)** LD decay analysis for the B‐sub‐genome; **(d)** LD decay analysis for the D‐sub‐genome.
**Supplementary Figure 2**. Manhattan plot for the: **(a)** shoot fresh weight (SFW; mg); **(b)** root length (RL; cm); **(c)** coleoptile length (CL; cm) under non‐heat stress (23°C) treatment.
**Supplementary Figure 3**. Manhattan plot for the: **(a)** shoot length (SL; cm); **(b)** root number (RN); **(c)** coleoptile length (CL; cm) under heat stress (36°C) treatment.
**Supplementary Figure 4**. Estimating the allelic effects of significant marker‐trait associations (MTAs) identified in the present study: **(a‐g)** allelic effects of MTAs, scaffold62159_9784648 (a), scaffold62159_9693200 (b), scaffold63259_2360029 (c), scaffold63259_2360398 (d), scaffold128405_6019031 (e), scaffold10127_4250444 (f), and scaffold4077_4635398 (g) identified under non‐heat stress (23°C) treatment; **(h‐w)** allelic effects of MTAs, scaffold468‐1_279183 (h), scaffold76743_1777606 (i), scaffold9496_2310787 (j), scaffold142879_1376183 (k), scaffold21647_3085029 (l), scaffold26910_6990976 (m), scaffold4982_9981508 (n), scaffold2159_4802103 (o), scaffold71558_289995 (p), scaffold5611_406983 (q), scaffold131852_2297774 (r), scaffold22822_3587896 (s), scaffold6791_22287451 (t), scaffold43799_1029672 (u), scaffold38798_23175 (v), and scaffold146879_1644623 (w) identified under heat stress (36°C) treatment.
