# Supplementary material for: Understanding the genetic basis of heat stress tolerance in wheat (Triticum aestivum L.) through genome‐wide association studies
Source: Plant Genome. 2025 Jul 9;18(3):e70071. doi: 10.1002/tpg2.70071 (PMC12241833; doi:10.1002/tpg2.70071)
Supplement: Supplementary file 1 [file TPG2-18-e70071-s001.docx]

**Supplementary Figure 1**. Analysis of linkage disequilibrium (LD) at the whole genome and sub-genome levels in the hexaploid spring wheat collection. **(a)** LD decay analysis of the whole genome; **(b)** LD decay analysis for the A-sub-genome; **(c)** LD decay analysis for the B-sub-genome; **(d)** LD decay analysis for the D-sub-genome.

**Supplementary Figure 2**. Manhattan plot for the: **(a)** shoot fresh weight (SFW; mg); **(b)** root length (RL; cm); **(c)** coleoptile length (CL; cm) under non-heat stress (23°C) treatment.

**Supplementary Figure 3**. Manhattan plot for the: **(a)** shoot length (SL; cm); **(b)** root number (RN); **(c)** coleoptile length (CL; cm) under heat stress (36°C) treatment.

**Supplementary Figure 4.** Estimating the allelic effects of significant marker-trait associations (MTAs) identified in the present study: **(a-g)** allelic effects of MTAs, scaffold62159_9784648 (a), scaffold62159_9693200 (b), scaffold63259_2360029 (c), scaffold63259_2360398 (d), scaffold128405_6019031 (e), scaffold10127_4250444 (f), and scaffold4077_4635398 (g) identified under non-heat stress (23°C) treatment; **(h-w)** allelic effects of MTAs, scaffold468-1_279183 (h), scaffold76743_1777606 (i), scaffold9496_2310787 (j), scaffold142879_1376183 (k), scaffold21647_3085029 (l), scaffold26910_6990976 (m), scaffold4982_9981508 (n), scaffold2159_4802103 (o), scaffold71558_289995 (p), scaffold5611_406983 (q), scaffold131852_2297774 (r), scaffold22822_3587896 (s), scaffold6791_22287451 (t), scaffold43799_1029672 (u), scaffold38798_23175 (v), and scaffold146879_1644623 (w) identified under heat stress (36°C) treatment.
